# Association between segmental and whole-body phase angle from bioelectrical impedance analysis and the physical function in community-dwelling older adults

**DOI:** 10.1038/s41598-026-44183-3

**Published:** 2026-06-17

**Authors:** Carmen Ayala-Martinez, Veronica Mihaiescu-Ion, Veronica Perez-Cabezas, Gloria Gonzalez-Medina, Beatriz Ortiz-Navarro, Margarita Carrillo de Albornoz-Gil, Alejandro Galan-Mercant

**Affiliations:** 1 Department of Nursing and Physical Therapy, Faculty of Nursing and Physical Therapy, University of Cadiz, Cadiz, Spain; 2 Department of Nursing and Physical Therapy, Faculty of Nursing and Physical Therapy, MOVE-IT Research Group, University of Cadiz, Cadiz, Spain; 3 Institute for Biomedical Research and Innovation of Cadiz (INIBICA), University of Cadiz, Cadiz, Spain; 4 Public Andalusian Health System, Málaga, Spain; 5 Sport and Physical Education Area, Faculty of Medicine, University of Malaga, Boulevard Louis Pasteur, Málaga, 29071 Spain

**Keywords:** Phase angle, Bioelectrical impedance analysis, Muscle quality, Segmental analysis, Physical function, Gait speed, Handgrip strength, Muscle power, Older adults, Frailty, Anatomy, Health care, Medical research, Physiology

## Abstract

Bioelectrical impedance analysis (BIA) and the derived phase angle (PhA) are recognized as a marker of cellular integrity and muscle quality, with emerging applications in the early detection of functional decline in older adults. However, evidence on the clinical utility of segmental PhA compared to whole-body measurements remains limited, particularly in community-dwelling populations. In contrast with previous Asian cohorts, this is the first study to systematically assess segmental and total PhA in a community-dwelling Spanish population. A cross-sectional study including 93 community-dwelling older adults aged ≥ 65 years was conducted in Southern Spain (median age: 75.5 years; 54.8% women and 45.2% men). Whole-body and segmental PhA (upper limbs, lower limbs, and trunk) were obtained using multifrequency BIA. Physical function was assessed using the Short Physical Performance Battery (SPPB), handgrip strength, gait speed (6-metre walk test), and lower-limb relative power (LL-rPOW). Multiple linear regression models adjusted by age, sex and BMI evaluated the associations between PhA and functional outcomes. Physical function outcomes (SPPB, LL-rPOW and Gait speed) showed numerically higher associations in an adjusted model with whole-body and lower-limb PhA. Lower-limb PhA demonstrated the highest association with SPPB (adjusted β = 0.538; *p* < 0.001) and was significantly associated with LL-rPOW (adjusted β = 0.356; *p* < 0.001) and gait speed (adjusted β = 0.335; *p* = 0.002). Upper-limb PhA had only significant association with handgrip strength (adjusted β = 0.220; *p* = 0.008). No significant associations were found for the PhA of the trunk. A clear anatomical specificity was observed, where the association strength of PhA increased when the anatomical region assessed matched the functional domain evaluated. Lower-limb PhA had higher numerically association with lower-extremity performance (SPPB, gait speed, LL-rPOW), whereas upper-limb PhA was primarily related to handgrip strength. Whole-body PhA was significantly associated with overall physical performance but lower-limb segmental PhA showed numerically higher standardized β values for lower-extremity outcomes, indicating greater regional specificity. These findings support segmental BIA as a rapid, non-invasive biomarker in primary care for stratification and monitoring functional status in community-dwelling older adults.

**Supplementary Information:**

The online version contains supplementary material available at 10.1038/s41598-026-44183-3.

## Introduction

The progressive ageing of the global population has led to an increased prevalence of geriatric syndromes, particularly sarcopenia and frailty, which are characterised by a decrease in skeletal muscle mass, strength, and physical function. These conditions are strongly associated with adverse health outcomes, including functional dependence, cognitive impairment, and increased mortality rates^1–3^. The World Health Organization has projected that by 2050, individuals aged 60 years and older will constitute approximately 22% of the global population^4,5^. Consequently, the identification of reliable, non-invasive, and early biomarkers to assess muscle cellular integrity and functional status in older adults has become a priority in clinical and research settings^6^, particularly within preventive healthcare domains such as primary care centres^7^.

In this context, Bioelectrical Impedance Analysis (BIA) has emerged as a widely used method for body composition assessment due to its portability, safety, and cost-effectiveness^8^. Beyond traditional body composition estimates, the Phase Angle (PhA), as a raw bioimpedance parameter, has gained significant attention as a superior indicator of cellular health^9–12^. PhA reflects cell membrane integrity and intracellular/extracellular water distribution, serving as a proxy for somatic protein mass and potentially muscle quality^9,13^. Unlike estimated muscle mass, which may remain stable despite functional decline, PhA has been shown to be a more sensitive marker for detecting early qualitative changes in muscle tissue that precede quantitative loss^13,14^.

Extensive literature supports the clinical relevance of PhA. Lower PhA values have been consistently observed in older adults with sarcopenia and frailty, correlated with reduced muscle strength and poor functional performance^10,12,15,16^. Furthermore, PhA has demonstrated prognostic value for mortality and hospitalisation risk in various clinical populations^11,17,18^ including advanced cancer^19^, critically ill ICU patients^20^, multimorbid older adults^21^, and has also been associated with neuromuscular performance in young athletes^22^. Specifically, reduced PhA has been associated with impairments in essential physical capabilities, such as gait speed, grip strength and the Short Physical Performance Battery (SPPB)^10,12,15,16^.

However, most of the existing research has focused on whole-body PhA measurements. Although valuable, whole-body values provide a systemic overview that can mask localised deficits in specific muscle groups, particularly in the lower extremities, which are crucial for mobility and balance. Recent evidence suggests that segmental PhA provides complementary physiological information to whole-body measurements by isolating specific body regions^18,22–24^. For instance, studies in athletic populations have indicated that regional PhA is more informative for monitoring neuromuscular performance than whole-body metrics^22^, and emerging research in hospitalized older adults suggests segmental analysis may enhance frailty detection^17^.

Muscle volume, muscle strength and physical performance decline with aging, but muscle strength decreases substantially faster than muscle mass^25^. The study of muscle performance should consider not only force but also shortening velocity, thus muscle power^26^. PhA is particularly relevant as it reflects “muscle quality” associated to sarcopenia – micro/macroscopic changes in muscle architecture and composition that precede quantitative mass loss^13^.Since most activities of daily living (ADL) require the capacity to support and mobilized one’s own body weight^3^, lower-limb relative muscle power (LL-rPOW) represents a critical but underexplored functional outcome. Lower-limb PhA should show particularly superior associations with lower-limb performance (SPPB, gait speed and LL-rPOW) because they are the primary contributors to locomotion and balance, while upper-limb PhA would most specifically relate to handgrip strength. Although substantial evidence from Asian cohorts, particularly Japanese populations^18,23,25,27^, has linked PhA to physical function, these findings may not be directly extrapolatable to Southern Spanish populations due to inherent differences in body composition, ethnicity, and hydration patterns. Furthermore, most studies focus on either whole-body PhA or isolated segments. Our study adds to the literature by providing a structured, head-to-head comparison of whole-body versus segmental PhA within the same dataset and by incorporating a novel, accessible measure of lower-limb relative power (LL-rPOW) derived from a smartphone application (PowerFrail)^28^, specifically tailored for primary care settings in Spain.

Therefore, the primary objective of this study was to evaluate and compare the association between whole-body and segmental phase angle across upper and lower extremities and trunk in relation to physical function by physical performance tests (SPPB, handgrip strength, gait speed and LL-rPOW) in a Spanish community-dwelling older population. We hypothesised that segmental PhA, particularly of the lower limbs, would demonstrate potentially enhanced associations with lower extremity function than whole-body PhA, and that anatomically congruent segment-function pairings would optimize the strength of these associations.

## Methods

### Study design and participants

An observational cross-sectional study was conducted. The final sample comprised 93 independent older adults, 42 men (45.2%) and 51 women (54.8%), with a median age of 75.5 year, living in their own homes and classified by geographic areas, therefore attending their corresponding primary care physicians, recruited from primary healthcare services of the Andalusian Health Service. All participants were capable of independent ambulation.

#### Inclusion criteria

- Older adults aged 65 years and over.
- Participants referred by their primary care physician.
- Barthel Index^29^ score > 95 points: functional independence in activities of daily living (ADL).
- Mini-Mental State Examination (MMSE)^30,31^ score ≥ 24: cognitively unimpaired to mild cognitive impairment. Ability to rise from and sit down on a chair independently. Signed informed consent.

#### Exclusion criteria

- Institutionalised participants.
- Individuals with implants or metallic materials that contraindicate bioelectrical impedance analysis.
- People with uncontrolled comorbidities or exacerbations of systemic disease in the past month.
- Individuals unable to adequately comply with study requirements for any reason, condition, or circumstance, at the investigator’s discretion.

#### Participant flow

**Fig. 1:**
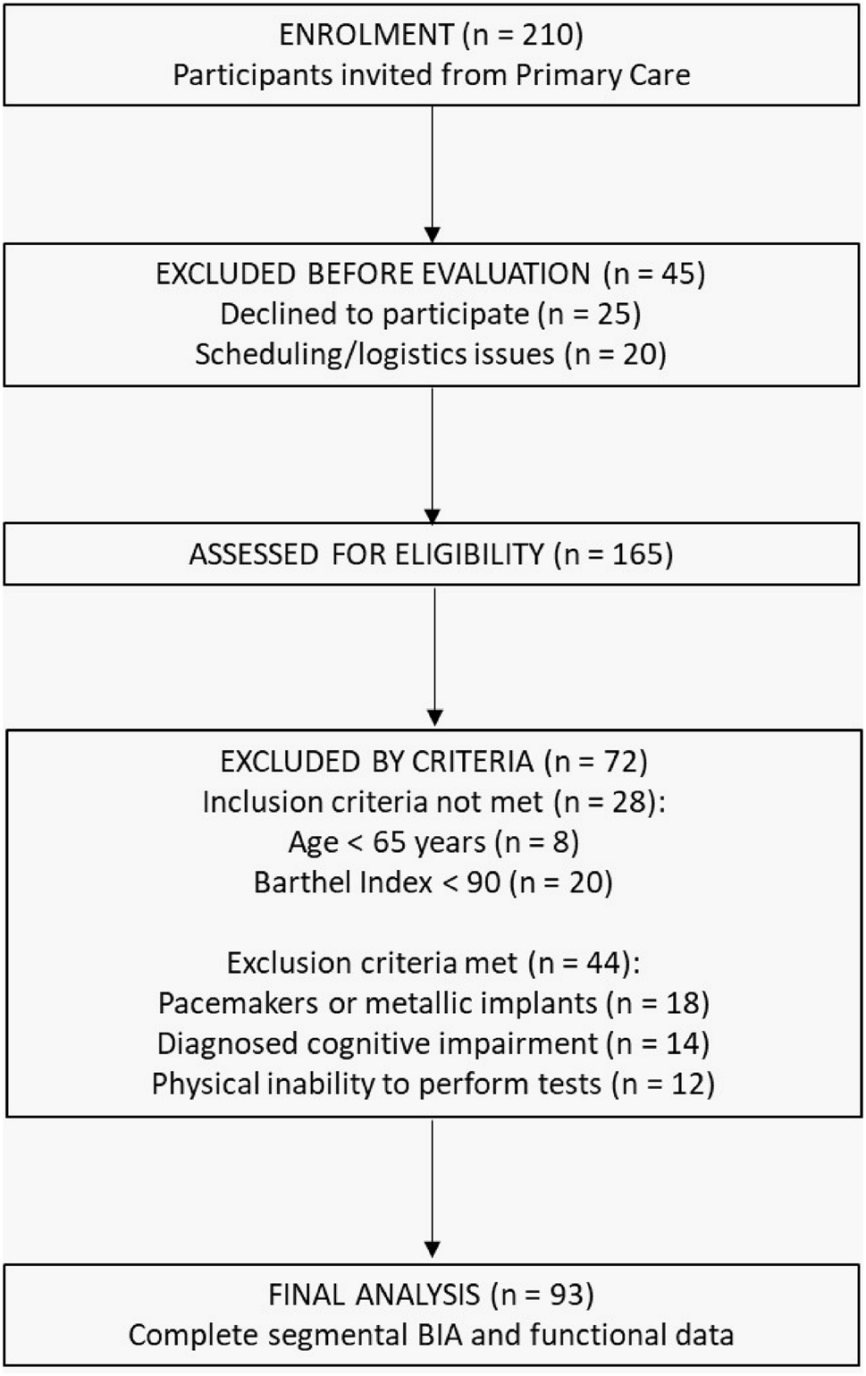
STROBE flow diagram of participant recruitment and selection process.

To improve transparency and reproducibility following the STROBE reporting guidelines for observational studies, Fig. 1 presents the participant flow diagram. From 210 participants referred by primary care physicians, 165 were assessed for eligibility, 72 were excluded based on inclusion/exclusion criteria, and 93 participants with complete segmental BIA and functional data were included in the final analysis. Detailed reasons for declinations prior to evaluation (*n* = 25) and exclusions after screening are provided.

### Procedures and measurements

The assessments were carried out in primary healthcare centres in Spain, facilities belonging to the Andalusian Health Service of different provinces. Participants were informed of the conditions for bioelectrical impedance analysis, instructed to refrain from vigorous physical exercise prior to assessment, and advised to attend the consultation in light, comfortable clothing without metallic objects. The recruitment was carried out during the second quarter of 2025. Participants were referred by their primary care physicians after confirmation that they met the inclusion criteria.

### Phase angle from bioelectrical impedance analysis

Phase angle and additional body composition parameters were assessed using multifrequency bioelectrical impedance analysis (BIA) with the InBody 770 device^32,33^. This device utilizes Direct Segmental Multi-Frequency BIA (DSM-MFBIA) technology, employing an eight-point tactile electrode system with hand-to-foot electrode placement to obtain direct impedance measurements at six different frequencies (1 kHz, 5 kHz, 50 kHz, 250 kHz, 500 kHz and 1000 kHz)^33^. The device performs 30 impedance measurements across five distinct body segments: left arm, right arm, left leg, right leg, and trunk, rather than treating the body as a single cylinder^33,34^.

Segmental PhA values, including trunk PhA, are derived automatically from the device algorithm, obtaining impedance measurements of each body segment. The trunk is measured independently because it constitutes approximately 50% fat-free mass, and separate measurement prevents substantial measurement error^34,35^. Upper and lower limb PhA values represent the mean of the left and right bilateral measurements^36^. All PhA values reported in this study are derived at 50 kHz, which is the standard frequency for phase angle assessment.

Phase angle is expressed in degrees and reflects the cellular health, intracellular/extracellular water distribution, and cell membrane integrity^9,13,14^. Regarding the bioelectrical impedance assessment protocol, participants were required to attend the evaluation after a minimum 3-hour fasting period and refraining from physical exercise on the day of the testing. Participants were instructed to wear light and comfortable clothing, without metal objects.

### Physical function

Physical function was evaluated through a comprehensive battery of physical performance tests, categorized into the Short Physical Performance Battery total score, a specific gait speed test, lower-limb relative power and handgrip strength.

*Short Physical Performance Battery (SPPB)*.

The Short Physical Performance Battery^37,38^, is a standardized and validated tool that evaluates lower extremity function across three key domains of mobility: static balance, gait speed, and lower limb strength through the sit-to-stand test. The SPPB yields a composite score ranging from 0 to 12 points, with lower scores representing poorer physical performance. All subtests were administered according to standardized protocol established by Guralnik et al^38^.:

- Static Balance Assessment (score range: 0–4 points): Participants were evaluated in three progressively challenging postural positions: side-by-side stand, semi-tandem stance, and tandem stance. Each position was held for up to 10 s. A hierarchical scoring system was applied based on the most challenging position successfully maintained: Score 0 (unable to hold side-by-side stand for 10 s); Score 1 (side-by-side stand held for 10 s only); Score 2 (semi-tandem stand held for 10 s, but tandem stand held for 0–2 s); Score 3 (full tandem stand held for 3–9 s); Score 4 (full tandem stand held for 10 s).
- Gait Speed (4-metre walk test; score range: 0–4 points): Usual gait speed was assessed over a 4-metre walking course^39^, unobstructed surface, following SPPB protocol^38,40^. Participants were instructed to walk at their usual pace and assistive devices were permitted if needed. The test was performed twice, and the fastest time was used for analysis. Gait speed over 4 m is a well-established indicator of mobility and risk of adverse outcomes in older adults^39,40^. Cut-points for the 4-metre walk were those proposed in SPPB: Score 0 (unable to complete); Score 1 (≥ 8.70 s); Score 2 (6.21–8.70 s); Score 3 (4.82–6.20 s); Score 4 (≤ 4.82 s).
- Sit-to-Stand Test (STS Test) (score range: 0–4 points): This subtest assessed the time required for participants to complete five repetitions of rising from and sitting down on a standard armless chair (placed next to a wall), with their arms across their chest. Timing was recorded from the initial sitting position to the final standing position after the fifth repetition. Time to complete five repetitions was classified according to quartiles: Score 0 (unable to complete); Score 1 (≥ 16.7 s); Score 2 (13.7–16.6 s); Score 3 (11.2–13.6 s); Score 4 (≤ 11.1 s).

### Relative Lower-limb muscle power/Lower-limb relative power (LL-rPOW)

LL-rPOW was assessed during the 5 repetitions sit-to-stand (5-STS) test using the PowerFrail mobile application installed on a smartphone (PowerFrail, Universidad de Castilla-La Mancha, version 1.0.10)^28^. The PowerFrail application uses video-based biomechanical analysis (NOT accelerometry or direct sensor signals). In our protocol, the smartphone camera recorded full-body sagittal plane movement, and the assessor manually selected the initial and final frames corresponding to the start and completion of the 5-STS and entered the total number of repetitions performed. Alternatively, the application also allows manual input of externally timed 5-STS tests using a stopwatch; however, only the video-based modality was used in the present study. The application then calculated relative lower-limb muscle power using the validated equation described by Alcázar et al.^41^,which incorporates the following variables: height and weight of the participants, chair height, number of repetitions completed, and total test execution time. Participants performed five consecutive sit-to-stand repetitions from a standardized armless chair (0.49 m height) in a controlled seated position with their buttocks touching the chair and arms crossed over their chest. Upon the standardised instruction “ready, set, go!“, participants performed STS repetitions as rapidly as possible, transitioning from the sitting position to full standing, with the test finishing when participants sat on the chair after the fifth STS repetition. LL-rPOW is considered a key indicator of functional status in older adults, as most activities of daily living (ADL) require the capacity to support and mobilise one’s own body weight^3^. The results are expressed in watts per kilogramme (W/kg), with higher values indicating greater relative muscle power.

### Gait speed using the 6-metre walk test

Participants were instructed to walk at their usual pace along a straight 6-metre walkway, with additional acceleration and deceleration zones of 1–2 m at the beginning and end to ensure that speed was captured during steady-state walking. Timing was initiated when the participant’s first foot crossed the 1-metre mark and stopped at the 7-metre point, following standardised procedures. The test was performed twice, and the fastest time (in seconds) was used to calculate the habitual gait speed (m/s). Usual gait speed is considered a robust indicator of mobility, frailty and risk of adverse outcomes such as disability, hospitalisation, and mortality, and is recommended as a core measure in geriatric assessment and primary care settings^42^. The 6-metre protocol was chosen over the 4-metre subtest of SPPB to assess usual gait speed, aligning with EWGSOP recommendations for sarcopenia screening^37^ and demonstrating strong prognostic value for adverse outcomes (e.g. speeds < 1.0 m/s identify older adults at high risk of persistent mobility limitation, hospitalization and mortality)^42^.

*Handgrip Strength test*.

Handgrip strength^37,43^ was assessed using a handheld dynamometer (Takei Physical Fitness Test adjustable dynamometer model), and results were expressed in kilogrammes (kg). Measurements were taken from the participant’s dominant hand^44,45^(defined as the self-reported hand used for writing and daily activities). Before testing, participants received brief standardised verbal instructions and a demonstration of the procedure.

Participants were positioned in an erect standing posture, with shoulders adducted and the arms fully extended, held parallel to the body, without touching the torso^44^. The dynamometer handle was adjusted to the participant’s hand size when necessary, following established protocols. Participants were instructed to exert maximum isometric force on the handle. Two trials were performed with 1-minute rest intervals between attempts, and the highest value of the two trials was recorded as the maximal handgrip strength (kg).

### Sample size calculation

The sample size was determined to ensure adequate power for the primary association analyses. Based on a significance level (*a*) of 0.05 and a statistical power (1-*b*) of 0.80, assuming a medium effect size (*f*^*2*^ = 0.15) in accordance with Cohen’s conventional benchmarks for multiple regression analyses^46^ and considering three main predictors (Segmental PhA, sex, and BMI), a minimum sample of 77 participants was required. Our final sample of 93 participants exceeded this threshold, providing a post-hoc power of over 90% for the main associations observed.

### Statistical analyses

The characteristics of the participants were described using descriptive statistics. Anthropometric and functional variables are presented as median and interquartile range (IQR), as the Shapiro–Wilk test revealed significant deviations from normality. To justify covariate selection, preliminary comparisons between-group by sex (men versus women) were conducted using the Mann-Whitney U test for independent samples, confirming significant differences. Associations between phase angle (whole body and segmental; independent variable) and physical function outcomes (handgrip strength, lower-limb relative power, SPPB score, and gait speed; dependent variables) were examined using linear regression analyses. For each physical function outcome, two models were constructed: (1) a crude model including only the phase angle variable of interest, and (2) an adjusted model controlling for age, sex, and body mass index (BMI), selected a priori based on their known association with muscle strength and physical function. However, lower-limb relative power (LL-rPOW) was adjusted only for age and sex, as weight is reflected in the calculation of relative power-strength (W/kg). Standardized (β) and unstandardized (B) coefficients with 95% confidence intervals were reported. Model fit was assessed using the coefficient of determination (R²). Statistical significance was set at *p* < 0.05. Data analysis was performed using JASP version 0.19.3 (JASP Team, 2025^47^).

### Ethics approval and consent to participate

This research was carried out according to the ethical standards established in the Declaration of Helsinki for human subject’s research. Before enrolment, all participants signed a written informed consent form acknowledging their voluntary participation. The study received ethical approval from the Institutional Andalusian Health Services Ethics Committee (protocol code: BON22). Privacy and confidentiality were always protected: all data were anonymised before analysis, and any identifying information was stored securely in a separate file from the research database. In this publication, no identifiable images or personal details of participants are presented. Participants received no financial or material compensation participating in the study.

### Results

**Fig. 2:**
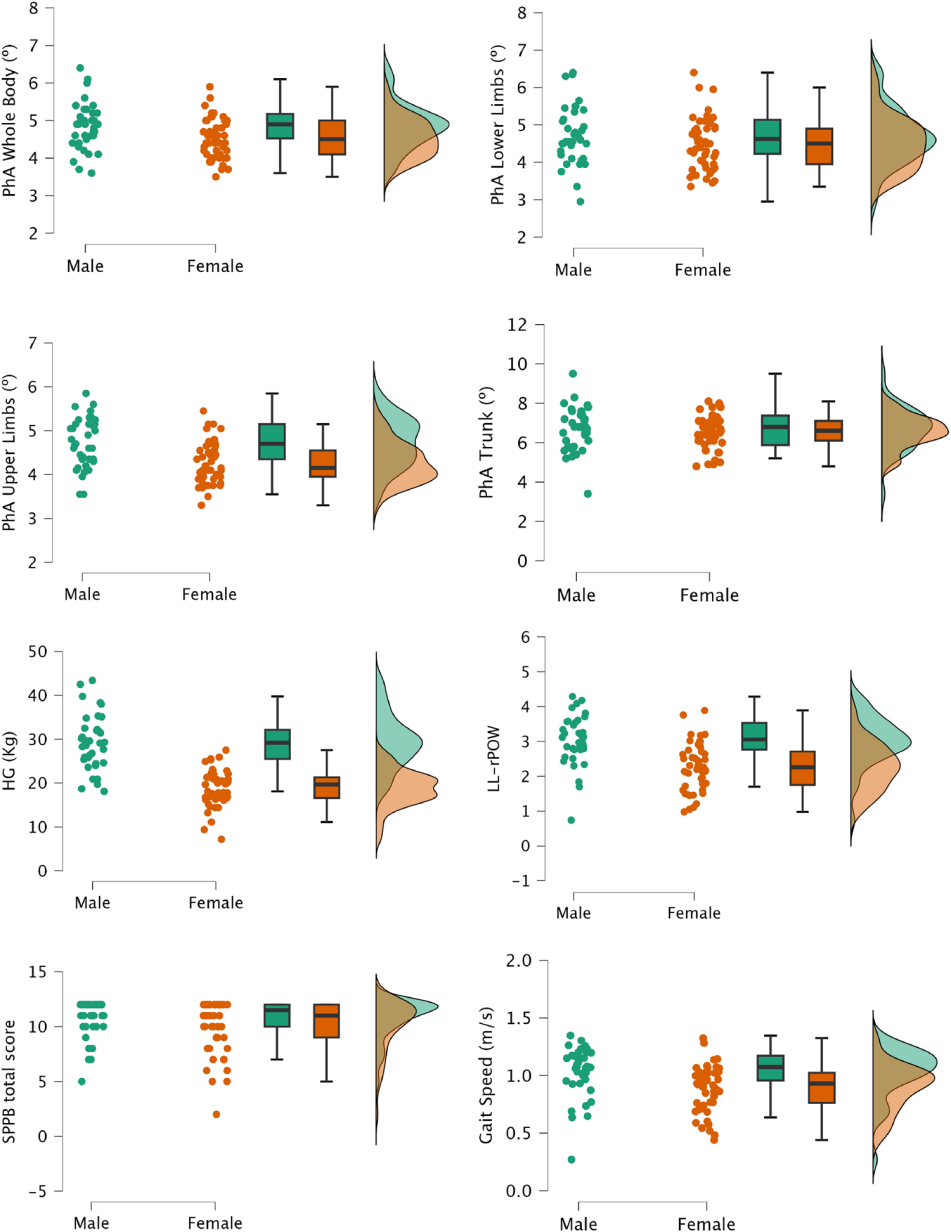
Sex differences in physical performance and bioelectrical impedance phase angle in older adults (*n* = 93).

The normality of the study variables was assessed using the Shapiro–Wilk test. Anthropometric and functional variables showed significant deviations from normality. Consequently, descriptive data are presented as median and interquartile range (IQR). A total of 93 community-dwelling older adults were included (median age: 75.5 years; 54.8% women (*n* = 51) and 45.2% men (*n* = 42)). The descriptive characteristics for the whole sample and stratified by sex are presented in Table 1 and illustrated in Fig. 2. Men were significantly taller and heavier than women (both *p* < 0.001). The whole-body phase angle and the upper-limb phase angle were also significantly higher in men compared to women (*p* = 0.007 and *p* < 0.001, respectively), while no significant sex differences were observed for the lower-limb or trunk phase angle. Regarding physical function, men exhibited significantly higher handgrip strength, lower-limb relative power (LL-rPOW), and gait speed (all *p* < 0.001). No significant differences were observed in the total SPPB score between sexes (*p* = 0.093). Body mass index did not differ significantly between men and women (*p* = 0.078).

**Table 1:** Descriptive characteristics of the study population in general and by sex (*n* = 93).

Variable	Total Sample Median (P25–P75)	Men (*n* = 42) Median (P25–P75)	Women (*n* = 51) Median (P25–P75)	U *p* - value
Age (years)	75.52 (72.88–77.21)	75.80 (73.33–78.39)	74.77 (72.28–76.69)	0.022
Height (cm)	161.20 (152.40–166.60)	167.10 (162.40–171.70)	152.90 (150.00–159.20)	< 0.001
Weight (kg)	70.00 (63.00–83.00)	78.10 (70.05–90.73)	67.60 (55.20–74.00)	< 0.001
BMI (kg/m²)	27.80 (24.85–32.30)	28.25 (25.33–33.10)	26.60 (24.10–30.70)	0.078
PhA Whole Body (°)	4.60 (4.20–5.00)	4.90 (4.53–5.18)	4.50 (4.10–5.00)	0.007
PhA Lower Limb (°)	4.55 (4.05–5.00)	4.63 (4.23–5.14)	4.50 (3.95–4.90)	0.070
PhA Upper Limb (°)	4.40 (4.05–4.85)	4.70 (4.35–5.15)	4.15 ( 3.95–4.55)	< 0.001
PhA Trunk (°)	6.70 (6.10–7.20)	6.80 (5.88–7.38)	6.60 (6.10–7.10)	0.195
Handgrip (kg)	22.10 (17.83–28.30)	29.10 (25.18–31.93)	19.67 (16.55–21.10)	< 0.001
LL-rPOW (W/kg)	2.70 (2.11–3.14)	2.98 (2.76–3.46)	2.30 (1.77–2.73)	< 0.001
SPPB (total score)	11.00 (10.00–12.00)	11.00 (10.00–12.00)	11.00 (9.00–12.00)	0.093
Gait Speed (m/s)	0.988 (0.769–1.078)	1.067 (0.927–1.163)	0.930 (0.764–1.024)	0.001

Values are presented as median (25th −75th percentile). SPPB total score is expressed in points (0–12). Group 1 = men; Group 2 = women. Between-group comparisons were performed using the Mann–Whitney U test.

Linear regression analyses were conducted to examine the association between segmental and whole-body phase angle (PhA) and different physical function outcomes, including handgrip strength, lower-limb relative power (LL-rPOW), Short Physical Performance Battery (SPPB), and gait speed. For each outcome, both crude and adjusted models (age, sex, and BMI) were calculated. Summary results are presented in Tables 2, 3, 4 and 5, the bold formatting was used to highlight key information and facilitate the reader’s identification of significant associations. Full multivariable models including all covariates are detailed in Supplementary Tables S1–S4. A relevant aspect of the present analyses is the difference observed between crude and adjusted models. In several outcomes, particularly for handgrip strength, some phase angle variables showed statistically significant associations in the crude model that disappeared after adjustment for age, sex, and BMI. This pattern suggests the presence of confounding effects, meaning that part of the apparent association in the unadjusted analysis was explained by demographic or anthropometric factors rather than by phase angle itself.

### Handgrip strength

As shown in Table 2, upper-limb PhA demonstrated higher numerically crude association with handgrip strength (β = 0.567, *p* < 0.001, R² = 0.322). After adjustment, this association remained statistically significant (β = 0.220, *p* = 0.008, R² = 0.640), whereas the associations observed for whole-body, lower-limb, and trunk PhA were attenuated and no longer significant. The complete adjusted models, including the contribution of sex, age, and BMI, are provided in Supplementary Table S1. In these models, sex and age consistently showed higher numerically associations with handgrip, while BMI displayed minimal influence.

**Table 2:** Linear Regression Analyses between Handgrip Strength and Phase Angle (*n* = 93).

PhA Variable	Model	β (Stand)	B (Unstand)	95% CI for B	*p*- value	*R*²
Whole Body PhA	Crude	0.422	5.262	2.825 to 7.699	< 0.001	0.178
	Adjusted*	0.118	1.471	−0.534 to 3.475	0.148	0.618†
Lower Limb PhA	Crude	0.289	2.875	0.820 to 4.930	0.007	0.083
	Adjusted*	0.043	0.423	−1.143 to 1.990	0.592	0.609†
Upper Limb PhA	Crude	0.567	7.398	5.082 to 9.713	< 0.001	0.322
	Adjusted*	**0.220**	**2.873**	**0.772 to 4.974**	**0.008**	**0.640†**
Trunk PhA	Crude	0.187	1.457	−0.190 to 3.105	0.082	0.035
	Adjusted*	0.071	0.551	−0.557 to 1.659	0.325	0.612†

The dependent variable in the model was Handgrip (kg), Phase Angle (°). * Adjusted for age, sex, and BMI. † R² corresponds to the full multivariable model including covariates. β = standardized coefficient; B = unstandardized coefficient; CI = confidence interval.

**Lower-Limb Relative Power (LL-rPOW)**.

In contrast to handgrip strength, LL-rPOW showed more signification in associations with whole-body and lower-limb PhA (Table 3). Both variables remained significantly associated after adjustment (β = 0.294 and β = 0.356; *p* = 0.004 and *p* < 0.001, respectively), whereas upper-limb and trunk PhA lost statistical significance. The detailed adjusted models (Supplementary Table S2) revealed that sex also contributed meaningfully to LL-rPOW variability, although the magnitude of association for lower-limb PhA remained substantial.

**Table 3:** Linear Regression Analyses between LL-rPOW and Phase Angle (*n* = 93).

Phase Variable	Model	β (Stand)	B (Unstand)	95% CI for B	*p*- value	*R*²
Whole Body PhA	Crude	0.476	0.642	0.387 to 0.898	< 0.001	0.227
	Adjusted*	0.294	0.397	0.127 to 0.666	0.004	0.387†
Lower Limb PhA	Crude	0.481	0.519	0.315 to 0.722	< 0.001	0.232
	Adjusted*	0.356	0.383	0.179 to 0.587	< 0.001	0.422†
Upper Limb PhA	Crude	0.340	0.479	0.193 to 0.766	0.001	0.115
	Adjusted*	0.041	0.058	−0.247 to 0.363	0.706	0.325†
Trunk PhA	Crude	0.204	0.172	−0.006 to 0.349	0.058	0.042
	Adjusted*	0.104	0.088	−0.067 to 0.242	0.263	0.334†

The dependent variable in the model was LL-rPOW (W/kg), Phase Angle (°). * Adjusted for age and sex. † R² corresponds to the full multivariable model including covariates. β = standardized coefficient; B = unstandardized coefficient; CI = confidence interval.

### Short physical performance battery (SPPB)

A similar pattern was observed for SPPB scores. As shown in Table 4, whole-body and lower-limb PhA presented moderate to higher numerically crude associations, which remained significant after adjustment (β = 0.444 and β = 0.538, respectively; both *p* < 0.001). Conversely, upper-limb and trunk PhA associations were attenuated and lost significance in adjusted models. Supplementary Table S3 details the full regression models, where BMI emerged as a consistent inverse factor across all SPPB analyses, while sex and age showed variable contributions depending on the segment analysed.

**Table 4:** Linear Regression Analyses between SPPB and Phase Angle (*n* = 93).

Phase Angle Variable	Model	β (Stand)	B (Unstand)	95% CI for B	*p*-value	*R*²
Whole Body PhA	Crude	0.435	1.585	0.878 to 2.293	< 0.001	0.189
	Adjusted*	0.444	1.616	0.868 to 2.364	< 0.001	0.377†
Lower Limb PhA	Crude	0.525	1.527	0.993 to 2.061	< 0.001	0.276
	Adjusted*	0.538	1.564	1.025 to 2.104	< 0.001	0.456†
Upper Limb PhA	Crude	0.229	0.874	0.074 to 1.674	0.033	0.053
	Adjusted*	0.161	0.615	−0.270 to 1.499	0.171	0.254†
Trunk PhA	Crude	0.219	0.498	0.019 to 0.976	0.042	0.048
	Adjusted*	0.103	0.234	−0.217 to 0.686	0.305	0.246†

The dependent variable in the model was SPBB, total score is expressed in points (0–12), Phase Angle (°). * Adjusted for age, sex, and BMI. † R² corresponds to the full multivariable model including covariates. β = standardized coefficient; B = unstandardized coefficient; CI = confidence interval.

### Gait speed

For gait speed, associations were again predominantly observed for lower-limb and whole-body PhA (Table 5). These relationships remained statistically significant after adjustment (β = 0.335 and β = 0.250, respectively), whereas trunk and upper-limb PhA did not demonstrate significant associations. The comprehensive adjusted models (Supplementary Table S4) indicated that age and BMI were consistently associated with slower gait speed, while sex effects were smaller in magnitude.

**Table 5:** Linear Regression Analyses between Gait Speed and Phase Angle (*n* = 93).

Phase Angle Variable	Model	β (Stand)	B (Unstand)	95% CI for B	*p*-value	*R*²
Whole Body PhA	Crude	0.354	0.133	0.057 to 0.209	< 0.001	0.125
	Adjusted*	0.250	0.094	0.012 to 0.175	0.024	0.306†
Lower Limb PhA	Crude	0.408	0.122	0.063 to 0.181	< 0.001	0.166
	Adjusted*	0.335	0.100	0.039 to 0.161	0.002	0.347†
Upper Limb PhA	Crude	0.201	0.079	−0.004 to 0.162	0.062	0.040
	Adjusted*	0.004	0.001	−0.089 to 0.092	0.975	0.262†
Trunk PhA	Crude	0.175	0.041	−0.009 to 0.091	0.104	0.031
	Adjusted*	0.052	0.012	−0.034 to 0.058	0.601	0.264†

The dependent variable in the model was Gait Speed (m/s), Phase Angle (°). * Adjusted for age, sex, and BMI. † R² corresponds to the full multivariable model including covariates. β = standardized coefficient; B = unstandardized coefficient; CI = confidence interval.

## Discussion

The present cross-sectional study investigated the association between different physical function tests (SPPB, LL-rPOW, gait speed, and handgrip strength) and PhA, assessed at both whole-body and segmental levels (upper limbs, lower limbs and trunk) in community-dwelling older adults from southern Spain. Our findings align with evidence previously reported in Asian cohorts, particularly Japanese populations^18,23,25,27^. However, given the distinct anthropometric and body composition profiles between Southern Spanish population and Asian cohorts, our findings in a Spanish primary care setting extend support for PhA as a biomarker of biological quality and cellular integrity across different ethnic backgrounds.

Furthermore, to the best of our knowledge, this is the first study to evaluate the association between relative lower-limb muscle power (LL-rPOW), derived from a smartphone application, and PhA, offering an accessible, low-cost tool for functional assessment in clinical practice. The main finding supports our hypothesis indicating a significant positive association between physical function and PhA, which appears to follow an anatomical specificity pattern. Lower-limb PhA showed numerically higher and more consistent associations with SPPB, LL-rPOW and gait speed, whereas upper-limb PhA showed a closer association with handgrip strength.

The only comparable experience identified in Spain is the recent study by Rivas-González et al. (2025)^17^, which evaluated segmental phase angle in relation to frailty detection in hospitalised older adults with cardiovascular disease. In contrast to this clinical setting, the present study provides novel evidence focused on healthy community-dwelling subjects. This distinction is clinically meaningful, as prior longitudinal studies have shown that lower PhA predicts frailty and other complex geriatric syndromes^48^ while, Yamada et al.^49^ reported that lower PhA values were associated with higher prevalence of both sarcopenia and dynapenia in older adults. Thus, this evidence supports the role of PhA as a physiological biomarker, complementary and non-invasive tool alongside established functional tests in primary care.

Our study systematically evaluated multiple, biomechanically specific functional tests (handgrip strength, SPPB, gait speed, and LL-rPOW) in relation to whole-body, upper-limb, lower-limb and trunk phase angle. The segment-specific approach contributes to a clearer physiological interpretation of the PhA–function relationships and describes these associations in community-dwelling older adults from southern Spain.Consistent with prior literature, our findings reinforce PhA as a indicator of muscle cellular integrity^6^, reflecting the ratio of intracellular to extracellular water and body cell mass density^13^. LowerPhA values have been associated withsarcopenia, increased disability risk^15,16,48^ and functional decline and thus loss of functional strength^23,48^. Recent investigations suggest that leg muscle cellular integrity, assessed by bioelectrical impedance analysis (BIA), is a critical determinant of gait speed and risk of falling^17,49,50^. Notably, Grootswagers et al.^51^ observed that in high-risk Dutch older adults significant crude Handgrip-PhA associations lost significance after adjustment for confounders (similar to our Table 2: Linear Regression Analyses between Handgrip Strength and Phase Angle).

When the different functional outcomes were examined jointly, a coherent segment-specific pattern of association emerged. Upper-limb phase angle was associated only with handgrip strength, whereas lower-limb and whole-body phase angle showed similar associations across tests mainly involving mobility and lower-extremity performance, including LL-rPOW, SPPB, and gait speed. In contrast, trunk phase angle demonstrated weak or non-significant associations after covariate adjustment. Overall, these findings suggest a functional correspondence between the anatomical segment from which phase angle is derived and the domain of physical performance assessed, highlighting the value of adjusted models to distinguish shared correlations from independent associations in the multifactorial context of health-related function. Additionally, an important aspect of these analyses is the difference between crude and adjusted models. In several outcomes, particularly handgrip strength, statistically significant crude associations lost significance after adjusting for age, sex, and BMI, indicating the presence of confounding effects whereby part of the initial association was explained by demographic or anthropometric factors rather than phase angle itself.

### Handgrip strength and phase angle

Handgrip strength showed a significant association with upper-limb PhA in the adjusted model (β = 0.220), whereas whole-body, lower-limb, and trunk PhA lost statistical significance after adjustment for age, sex, and BMI. The observed pattern may indicate a degree of anatomical specificity, given that only the PhA from the upper extremities was significantly associated with arm strength.The marked decrease in model fit (β from 0.567 crude to 0.220 adjusted) further suggests that sex and age are important confounders in this relationship, consistent with their known influence on upper-body strength in older adults. These findings imply that upper-limb PhA could offer segment-specific insights that complement whole-body measurements in evaluating upper-extremity muscle function.

### LL-rPOW and phase angle

A novel and central finding of our study is that lower-limb relative power (LL-rPOW) was significantly associated with both whole-body PhA (β = 0.294) and lower-limb PhA (β = 0.356) in the adjusted models (age and sex only as confounders variables). Notably, lower-limb PhA showed numerically higher model fit compared to whole-body PhA, while upper-limb and trunk PhA were non-significant. This anatomical correspondence between lower-limb PhA and lower-limb relative power (a key functional outcome for activities of daily living) supports the hypothesis that segmental analysis provides physiologically relevant information for lower-extremity performance. The absence of BMI adjustment in these models avoids mathematical coupling, as body mass is already accounted for in the W/kg normalization.

### SPPB and phase angle

The Short Physical Performance Battery (SPPB) total score showed statistically significant associations with whole-body PhA (β = 0.444) and lower-limb PhA (β = 0.538) after adjustment for age, sex and BMI. Lower-limb PhA exhibited numerically higher model fit than whole-body PhA, whereas upper-limb and trunk PhA were non-significant. Since SPPB aggregates performance across three distinct but complementary domains (static balance, lower-limb strength, and gait speed), it provides a comprehensive segmental assessment of functional capacity. Consequently, it is biologically plausible that the specific bioelectrical status of the legs, which constitutes the common physiological substrate required for all these tasks, is more closely associated with this composite score than any single functional metric alone. This pattern appears to reflect anatomical specificity in segmental PhA, suggesting its potential value as a complementary measure of mobility performance in older adults.

### Gait speed and phase angle

Gait speed showed statistically significant associations with whole-body and lower-limb PhA (β = 0.250 and β = 0.335respectively) in the adjusted models. Lower-limb PhA demonstrated numerically higher model fit compared to whole-body PhA, while upper-limb and trunk PhA were non-significant. Given that gait speed is a robust predictor of adverse outcomes in older adults^42^, these findings lend support to the view that lower-limb PhA may represent a complementary physiological biomarker for mobility assessment in primary care and community settings.

### Lack of association in trunk PhA

Unlike the extremities, we found no significant associations between trunk PhA and any functional variable. This can be explained by the anatomical characteristics of the trunk, which contains a large proportion of visceral organs and fluids that do not contribute to force generation but influence impedance^6^. The literature reports that the trunk PhA is less sensitive to changes in skeletal muscle mass compared to the limbs due to its large cross-sectional area and low resistance^52,53^. Therefore, we suggest that for functional assessment purposes in older adults, the trunk PhA could have limited clinical utility.

### Clinical implications: towards precision medicine

A key clinical implication of our findings is the potential of segmental BIA to facilitate more precise and individualised interventions, bringing us closer to precision medicine in the management of physical function in older adults. Standard whole-body evaluation might mask specific regional deficits; for example, a patient might have a preserved whole-body PhA but a critically low lower-limb PhA, placing them at occult risk of falls. In such a scenario, a general exercise prescription might be less effective than a targeted intervention focusing specifically on lower-body resistance and balance training. As highlighted in recent reviews on geriatric syndromes, biomarkers that allow risk stratification and individualized care strategies are urgently needed^10,12^. By identifying which specific body segment is compromised (e.g., upper vs. lower limbs), clinicians can tailor rehabilitation programmes to the specific bioelectrical deficit, potentially improving the efficiency and outcomes of physical therapies. The segment-specific approach may highlight differential associations that the conventional “one-size-fits-all” strategy could overlook, though these potential advantages should be interpreted cautiously given the cross-sectional design.

## Limitations and conclusion

The strengths of this study include the stratified analysis by body segments, allowing a more nuanced understanding of the PhA-function relationship in a Spanish population. However, several limitations should be acknowledged. The cross-sectional design precludes any inference of causality. Although the sample size was sufficient to detect statistically significant associations, larger and more heterogeneous cohorts would be desirable to confirm these findings. Finally, the population was limited to older adults from Southern Spain, which may limit the generalisability of results to other populations and ethnic groups.

In addition, the relatively low PhA values and modest physical function scores observed even among community-dwelling participants may reflect the inclusion of a comparatively frail sample, potentially influencing the magnitude of the associations detected. The set of covariates used in the adjusted models was limited, and residual confounding by unmeasured variables cannot be ruled out.

In conclusion, physical function showed significant associations with PhA in older adults with numerically higher model fit observed using anatomically congruent segmental PhA (Tables 2, 3, 4 and 5). Our findings demonstrate that lower-limb PhA is more associated with SPPB, lower-limb relative power, and gait speed, while upper-limb PhA is more closely related to handgrip strength. These cross-sectional findings suggest that segmental bioimpedance could provide complementary physiological information alongside functional tests in primary care. However, while these cross-sectional findings are promising, longitudinal studies are required to confirm its predictive validity and clinical utility before recommending its routine implementation in geriatric populations.

## Supplementary Information

Below is the link to the electronic supplementary material.

Supplementary Material 1

## Data Availability

The clinical datasets that support the findings of this study are available from the Servicio Andaluz de Salud (SAS), but restrictions apply to the availability of these data, which were used under license for the current study and so are not publicly available due to data protection and privacy regulations. The data are, however, available from the authors upon reasonable request and with the permission of the Servicio Andaluz de Salud (SAS) and the corresponding Research Ethics Committee.
