# Supplementary material for: Association between segmental and whole-body phase angle from bioelectrical impedance analysis and the physical function in community-dwelling older adults
Source: Sci Rep. 2026 Jun 17;16:18853. doi: 10.1038/s41598-026-44183-3 (PMC13276190; doi:10.1038/s41598-026-44183-3)
Supplement: Supplementary file 1 — Supplementary Material 1 [file 41598_2026_44183_MOESM1_ESM.docx]

**Supplementary Table S1.** Full Multivariable Linear Regression Models for Handgrip Strength (n = 93)

**S1A.** Whole Body Phase Angle

| **Variable** | **B** | **SE** | **β** | **95% CI** | **p** |
| --- | --- | --- | --- | --- | --- |
| **Intercept** | 68.094 | 18.039 |  | 32.208 – 103.980 | **<0.001** |
| **Sex** | −10.514 | 1.102 |  | −12.707 – −8.320 | **<0.001** |
| **BMI** | 0.048 | 0.097 | 0.036 | −0.145 – 0.241 | 0.622 |
| **Age** | −0.626 | 0.198 | −0.249 | −1.020 – −0.233 | **0.002** |
| **PhA Whole Body** | 1.471 | 1.008 | 0.118 | −0.534 – 3.475 | 0.148 |

**Model R² = 0.618**

**S1B.** Lower Limb Phase Angle

| **Variable** | **B** | **SE** | **β** | **95% CI** | **p** |
| --- | --- | --- | --- | --- | --- |
| **Intercept** | 78.453 | 17.701 |  | 43.241 – 113.665 | **<0.001** |
| **Sex** | −10.936 | 1.076 |  | −13.075 – −8.796 | **<0.001** |
| **BMI** | 0.065 | 0.098 | 0.048 | −0.129 – 0.259 | 0.508 |
| **Age** | −0.702 | 0.201 | −0.279 | −1.101 – −0.303 | **<0.001** |
| **PhA Lower Limb** | 0.423 | 0.787 | 0.043 | −1.143 – 1.990 | 0.592 |

**Model R² = 0.609**

**S1C.** Upper Limb Phase Angle

| **Variable** | **B** | **SE** | **β** | **95% CI** | **p** |
| --- | --- | --- | --- | --- | --- |
| **Intercept** | 57.594 | 17.030 |  | 23.716 – 91.472 | **0.001** |
| **Sex** | −9.611 | 1.138 |  | −11.875 – −7.348 | **<0.001** |
| **BMI** | 0.025 | 0.095 | 0.019 | −0.163 – 0.213 | 0.791 |
| **Age** | −0.564 | 0.187 | −0.224 | −0.935 – −0.193 | **0.003** |
| **PhA Upper Limb** | 2.873 | 1.056 | 0.220 | 0.772 – 4.974 | **0.008** |

**Model R² = 0.640**

**S1D.** Trunk Phase Angle

| **Variable** | **B** | **SE** | **β** | **95% CI** | **p** |
| --- | --- | --- | --- | --- | --- |
| **Intercept** | 76.105 | 16.522 |  | 43.238 – 108.972 | **<0.001** |
| **Sex** | −10.918 | 1.053 |  | −13.012 – −8.823 | **<0.001** |
| **BMI** | 0.083 | 0.098 | 0.062 | −0.111 – 0.277 | 0.395 |
| **Age** | −0.701 | 0.187 | −0.279 | −1.072 – −0.329 | **<0.001** |
| **PhA Trunk** | 0.551 | 0.557 | 0.071 | −0.557 – 1.659 | 0.325 |

**Model R² = 0.612**

**Note: B** = unstandardized coefficient; **β** = standardized coefficient; **SE** = standard error; **CI** = confidence interval.

**Supplementary Table S2.** Full Multivariable Linear Regression Models for LL-rPOW (n = 93)

**S2A.** Whole Body Phase Angle

| **Variable** | **B** | **SE** | **β** | **95% CI** | **p** |
| --- | --- | --- | --- | --- | --- |
| **Intercept** | 4.381 | 2.390 |  | −0.372 – 9.135 | 0.070 |
| **Sex** | −0.693 | 0.149 |  | −0.989 – −0.397 | **<0.001** |
| **Age** | −0.043 | 0.027 | −0.159 | −0.096 – 0.010 | 0.109 |
| **PhA Whole Body** | 0.397 | 0.136 | 0.294 | 0.127 – 0.666 | **0.004** |

**Model R² = 0.387**

**S2B.** Lower Limb Phase Angle

| **Variable** | **B** | **SE** | **β** | **95% CI** | **p** |
| --- | --- | --- | --- | --- | --- |
| **Intercept** | 3.832 | 2.225 |  | −0.594 – 8.258 | 0.089 |
| **Sex** | −0.723 | 0.139 |  | −0.999 – −0.448 | **<0.001** |
| **Age** | −0.034 | 0.026 | −0.127 | −0.086 – 0.017 | 0.187 |
| **PhA Lower Limb** | 0.383 | 0.102 | 0.356 | 0.179 – 0.587 | **<0.001** |

**Model R² = 0.422**

**S2C.** Upper Limb Phase Angle

| **Variable** | **B** | **SE** | **β** | **95% CI** | **p** |
| --- | --- | --- | --- | --- | --- |
| **Intercept** | 8.410 | 2.439 |  | 3.559 – 13.262 | **<0.001** |
| **Sex** | −0.826 | 0.167 |  | −1.158 – −0.494 | **<0.001** |
| **Age** | −0.075 | 0.027 | −0.275 | −0.129 – −0.021 | **0.007** |
| **PhA Upper Limb** | 0.058 | 0.153 | 0.041 | −0.247 – 0.363 | 0.706 |

**Model R² = 0.325**

**S2D.** Trunk Phase Angle

| **Variable** | **B** | **SE** | **β** | **95% CI** | **p** |
| --- | --- | --- | --- | --- | --- |
| **Intercept** | 7.898 | 2.111 |  | 3.700 – 12.096 | **<0.001** |
| **Sex** | −0.837 | 0.145 |  | −1.125 – −0.549 | **<0.001** |
| **Age** | −0.072 | 0.025 | −0.265 | −0.123 – −0.021 | **0.006** |
| **PhA Trunk** | 0.088 | 0.078 | 0.104 | −0.067 – 0.242 | 0.263 |

**Model R² = 0.334**

**Note: B** = unstandardized coefficient; **β** = standardized coefficient; **SE** = standard error; **CI** = confidence interval.

**Supplementary Table S3.** Full Multivariable Linear Regression Models for SPPB Total Score (n = 93)

**S3A.** Whole Body Phase Angle

| **Variable** | **B** | **SE** | **β** | **95% CI** | **p** |
| --- | --- | --- | --- | --- | --- |
| **Intercept** | 17.777 | 6.734 |  | 4.381 – 31.173 | **0.010** |
| **Sex** | −0.708 | 0.412 |  | −1.526 – 0.111 | 0.089 |
| **BMI** | −0174 | 0.036 | −0.440 | −0.246 – −0.101 | **<0.001** |
| **Age** | −0.128 | 0.074 | −0.175 | −0.275 – 0.019 | 0.086 |
| **PhA Whole Body** | 1.616 | 0.376 | 0.444 | 0.868 – 2.364 | **<0.001** |

**Model R² = 0.377**

**S3B.** Lower Limb Phase Angle

| **Variable** | **B** | **SE** | **β** | **95% CI** | **p** |
| --- | --- | --- | --- | --- | --- |
| **Intercept** | 15.262 | 6.102 |  | 3.123 – 27.400 | **0.014** |
| **Sex** | −0.820 | 0.371 |  | −1.558 – −0.082 | **0.030** |
| **BMI** | −0.170 | 0.034 | −0.431 | −0.237 – −0.103 | **<0.001** |
| **Age** | −0.091 | 0.069 | −0.123 | −0.228 – 0.047 | 0.194 |
| **PhA Lower Limb** | 1.564 | 0.271 | 0.538 | 1.025 – 2.104 | **<0.001** |

**Model R² = 0.456**

**S3C.** Upper Limb Phase Angle

| **Variable** | **B** | **SE** | **β** | **95% CI** | **p** |
| --- | --- | --- | --- | --- | --- |
| **Intercept** | 29.435 | 7.170 |  | 15.170 – 43.699 | **<0.001** |
| **Sex** | −1.008 | 0.479 |  | −1.961 – −0.055 | **0.038** |
| **BMI** | −0.159 | 0.040 | −0.402 | −0.238 – −0.079 | **<0.001** |
| **Age** | −0.223 | 0.079 | −0.304 | −0.379 – −0.067 | **0.006** |
| **PhA Upper Limb** | 0.615 | 0.445 | 0.161 | −0.270 – 1.499 | 0.171 |

**Model R² = 0.254**

**S3D.** Trunk Phase Angle

| **Variable** | **B** | **SE** | **β** | **95% CI** | **p** |
| --- | --- | --- | --- | --- | --- |
| **Intercept** | 31.769 | 6.736 |  | 18.369 – 45.169 | **<0.001** |
| **Sex** | −1.255 | 0.429 |  | −2.109 – −0.401 | **0.004** |
| **BMI** | −0.143 | 0.040 | −0.364 | −0.223 – −0.064 | **<0.001** |
| **Age** | −0.242 | 0.076 | −0.330 | −0.394 – −0.091 | **0.002** |
| **PhA Trunk** | 0.234 | 0.227 | 0.103 | −0.217 – 0.686 | 0.305 |

**Model R² = 0.246**

**Note: B** = unstandardized coefficient; **β** = standardized coefficient; **SE** = standard error; **CI** = confidence interval.

**Supplementary Table S4.** Full Multivariable Linear Regression Models for Gait Speed (n = 93)

**S4A.** Whole Body Phase Angle

| **Variable** | **B** | **SE** | **β** | **95% CI** | **p** |
| --- | --- | --- | --- | --- | --- |
| **Intercept** | 2.395 | 0.732 |  | 0.939 – 3.851 | **0.002** |
| **Sex** | −0.155 | 0.045 |  | −0.244 – −0.066 | **<0.001** |
| **BMI** | −0.013 | 0.004 | −0.324 | −0.021 – −0.005 | **0.001** |
| **Age** | −0.019 | 0.008 | −0.250 | −0.035 – −0.003 | **0.021** |
| **PhA Whole Body** | 0.094 | 0.041 | 0.250 | 0.012 – 0.175 | **0.024** |

**Model R² = 0.306**

**S4B.** Lower Limb Phase Angle

| **Variable** | **B** | **SE** | **β** | **95% CI** | **p** |
| --- | --- | --- | --- | --- | --- |
| **Intercept** | 2.127 | 0.689 |  | 0.756 – 3.498 | **0.003** |
| **Sex** | −0.159 | 0.042 |  | −0.242 – −0.075 | **<0.001** |
| **BMI** | −0.013 | 0.004 | −0.322 | −0.021 – −0.006 | **<0.001** |
| **Age** | −0.016 | 0.008 | −0.207 | −0.031 – −0.000 | **0.048** |
| **PhA Lower Limb** | 0.100 | 0.031 | 0.335 | 0.039 – 0.161 | **0.002** |

**Model R² = 0.347**

**S4C.** Upper Limb Phase Angle

| **Variable** | **B** | **SE** | **β** | **95% CI** | **p** |
| --- | --- | --- | --- | --- | --- |
| **Intercept** | 3.384 | 0.735 | — | 1.922 – 4.845 | **<0.001** |
| **Sex** | −0.190 | 0.049 | — | −0.288 – −0.092 | **<0.001** |
| **BMI** | −0.012 | 0.004 | −0.290 | −0.020 – −0.004 | **0.005** |
| **Age** | −0.027 | 0.008 | −0.352 | −0.043 – −0.011 | **0.001** |
| **PhA Upper Limb** | 0.001 | 0.046 | 0.004 | −0.089 – 0.092 | 0.975 |

**Model R² = 0.262**

**S4D.** Trunk Phase Angle

| **Variable** | **B** | **SE** | **β** | **95% CI** | **p** |
| --- | --- | --- | --- | --- | --- |
| **Intercept** | 3.227 | 0.686 | — | 1.863 – 4.591 | **<0.001** |
| **Sex** | −0.187 | 0.044 | — | −0.274 – −0.100 | **<0.001** |
| **BMI** | −0.011 | 0.004 | −0.282 | −0.020 – −0.003 | **0.006** |
| **Age** | −0.026 | 0.008 | −0.339 | −0.041 – −0.010 | **0.001** |
| **PhA Trunk** | 0.012 | 0.023 | 0.052 | −0.034 – 0.058 | 0.601 |

**Model R² = 0.264**

**Note: B** = unstandardized coefficient; **β** = standardized coefficient; **SE** = standard error; **CI** = confidence interval.
